# Biodiesel Exhaust: The Need for Health Effects Research

**DOI:** 10.1289/ehp.9631

**Published:** 2007-01-03

**Authors:** Kimberly J. Swanson, Michael C. Madden, Andrew J. Ghio

**Affiliations:** 1 Department of Environmental Sciences and Engineering, School of Public Health, University of North Carolina, Chapel Hill, North Carolina, USA; 2 National Health and Environmental Effects Research Laboratory, U.S. Environmental Protection Agency, Research Triangle Park, North Carolina, USA

**Keywords:** air pollution, biodiesel, diesel exhaust, diesel fuels, lung diseases, vehicle emissions

## Abstract

**Background:**

Biodiesel is a diesel fuel alternative that has shown potential of becoming a commercially accepted part of the United States’ energy infrastructure. In November 2004, the signing of the Jobs Creation Bill HR 4520 marked an important turning point for the future production of biodiesel in the United States because it offers a federal excise tax credit. By the end of 2005, industry production was 75 million gallons, a 300% increase in 1 year. Current industry capacity, however, stands at just over 300 million gallons/year, and current expansion and new plant construction could double the industry’s capacity within a few years. Biodiesel exhaust emission has been extensively characterized under field and laboratory conditions, but there have been limited cytotoxicity and mutagenicity studies on the effects of biodiesel exhaust in biologic systems.

**Objectives:**

We reviewed pertinent medical literature and addressed recommendations on testing specific research needs in the field of biodiesel toxicity.

**Discussion:**

Employment of biodiesel fuel is favorably viewed, and there are suggestions that its exhaust emissions are less likely to present any risk to human health relative to petroleum diesel emissions.

**Conclusion:**

The speculative nature of a reduction in health effects based on chemical composition of biodiesel exhaust needs to be followed up with investigations in biologic systems.

The use of plant oils as fuels in the compression ignition engine is as old as the engine itself (Knothe 2001). Although vegetable oil alone proved too viscous for continuous use in the diesel engine, plant oil–based fuels were developed during the early part of the 20th century (Knothe 2005). Biodiesel today describes an alkyl ester of oils that can be used in an unmodified diesel engine. Feedstocks used in the production of biodiesel are renewable sources of biologic origin. Although animal oils can be used to produce biodiesel, plant oils are more abundant and represent most of the feedstock used in biodiesel production on a commercial scale in the United States. Currently, the primary plant oil feedstocks for the United States and Europe are soybean oil and rapeseed oil, respectively. The fatty acids in plant triacylglycerols contain unsaturated carbon bonds and thus tend to remain in liquid form at low temperatures. These liquid oils currently require a modified diesel engine, limiting their usefulness as an alternative fuel source at this time. Chemical modification of the plant oil is required to produce a fuel that can be used in an unmodified diesel engine. Base-catalyzed transesterification is the current industrial reaction method for biodiesel (Figure 1). This process reacts a plant oil with an alcohol (i.e., methanol) in the presence of a catalyst [e.g., potassium hydroxide (KOH)] to produce an alcohol ester (i.e., methyl ester) and glycerol (Schuchardt et al. 1998). Alkylated fatty acids are less dense than glycerol and therefore separate on the basis of density. The glycerol by-product is removed from the bottom of the reaction vessel. The glycerol can be further refined for use in the pharmaceutical and cosmetic industries. This type of reaction is performed as a batch process which can be scaled up depending on the capacity and design of the reactor.

Biodiesel production and use in the United States began in the 1970s. Cooperatives and small batch producers have provided a limited supply of fuel to a small number of cars and farm equipment. Production is now increasing. Biodiesel production in 2004 was approximately 25 million gallons (Figure 2). The enactment of the American Jobs Creation Act of 2004 marked an important turning point for the future production of biodiesel in the United States. At the heart of the bill is a provision that allows distributors of biodiesel and biodiesel blends (mixed with petroleum diesel) to receive a federal excise tax credit. There is now a greater economic incentive for increasing biodiesel production in the United States. By the end of 2005, industry production was 75 million gallons, a 300% increase in 1 year. Current industry capacity, however, stands at just over 300 million gallons/year; expansion and new plant construction could double the industry’s capacity within a few years.

The transportation sector was the first to adopt biodiesel as a competing fuel alternative. Growing numbers of federal and municipal fleets across the country have turned to biodiesel as the preferred fuel, demonstrating a commitment of public officials to environmental stewardship. Other sectors that are technically capable of using biodiesel include marine transportation, residential heating, and mining. However, with the industry operating at peak production, it is estimated that biodiesel will meet < 1.0% of current diesel demand (Swanson 2006). Clearly, biodiesel will not be replacing petroleum diesel in terms of usage in the foreseeable future.

## Composition of Biodiesel Exhaust Emissions

Relative to petroleum diesel emissions, biodiesel emissions have been shown to contain less particulate matter, carbon monoxide, and polycyclic aromatic hydrocarbons (PAHs) (Graboski et al. 2003; McDonald and Spears 1997; Sharp et al. 2000). Furthermore, sulfur-containing compounds appear to be undetectable. However, the combustion of biodiesel in a diesel engine typically does increase the release in nitrogen oxides, which, in addition to inducing potential health effects, have been identified as an ozone precursor. Additionally, despite the reduction in a total mass of particulate matter, the soluble organic fraction of the emitted particles is commonly a greater percentage of biodiesel exhaust emissions, whereas a smaller percentage of insoluble mass is present relative to petroleum diesel soot (Durbin et al. 1999). One investigation has demonstrated a 30% decrease in particulate emissions with use of 100% biodiesel, but the soluble organic fraction increased by roughly 40% (Sharp 1998). This smaller production of particles with a greater concentration of soluble organic fraction may impact the biologic effects and toxicity of biodiesel exhaust particles. The U.S. Environmental Protection Agency (EPA) has enacted a series of emission standards for various on-road and off-road diesel engine types that will be put into place over approximately the next 10 years (U.S. EPA 2004). These standards are intended to reduce specific components of diesel exhaust (i.e., nitrogen oxide and particulate matter concentrations) through various strategies such as decreased fuel sulfur content and particle traps; however, it is unclear how much decrease (if any) will be observed for many gas-phase components derived from petroleum and biodiesel combustion.

The U.S. EPA in 2002 released a draft technical report (EPA420-P-02-001) of a comprehensive analysis of the emission impacts of biodiesel using publicly available data (U.S. EPA 2002a). Statistical regression analysis correlated the concentration of biodiesel in mixtures of conventional diesel fuel with changes in regulated and unregulated pollutants emitted from on-road heavy-duty diesel engines. A selection of mobile source air toxics was included in the analysis of biodiesel’s effects on emission. These are referred to as the “aggregated toxics” (i.e., acetaldehyde, acrolein, benzene, 1,3-butadiene, ethylbenzene, *n*-hexane, naphthalene, styrene, toluene, and xylene); most are currently unregulated. Studies used in the analysis offered limited data on toxics detected in biodiesel exhaust, but this analysis revealed that the mass ratio of total toxics to hydrocarbons actually increased with the addition of biodiesel. In other words, although the total hydrocarbon measurement decreased, there was a shift in the composition toward more unregulated pollutants. This preliminary analysis, however, showed that this shift was not large enough to cause total toxic emissions to increase with biodiesel use compared to conventional diesel fuel.

Similar to other combustion emissions, biodiesel exhaust emissions contain irritant gases and vapors such as nitrogen oxides, aldehydes, and a wide range of organic compounds, some of which are also likely to present an oxidative stress (Mauderly 1997). The potential of biodiesel to form aldehydes is related to fuel quality. When used in a diesel engine, biodiesel with a high glycerol content (indicating poor post-transesterification refining) produces emissions with increased acrolein levels (Graboski and McCormick 1998). Ethanol and methanol—typical alcohols used in biodiesel production—are aldehyde precursors, and if not removed from the biodiesel could combust partially to form formaldehyde and acetaldehyde which are classified as a carcinogen and possible carcinogen, respectively [International Agency for Research on Cancer (IARC) 1985; McDonald and Spears 1997].

## Biodiesel Exhaust Emissions and Human Health

There is insufficient information regarding the mutagenic potential of biodiesel emissions specific to fuel category (e.g., starting oil feed-stock), engine type, and operating conditions. In *in vitro* bacterial assays, most of the mutagenic activity in biodiesel exhaust is contributed by a minority of the soluble organic fraction mass, particularly PAHs (Mauderly 1997). To date only one published *in vitro* study has evaluated mutagenicity of biodiesel soluble organic fraction using a competent mammalian cell model (rat hepatocytes) in addition to the traditional Ames assay (Eckl et al. 1997). The authors observed in the Ames assay higher mutagenic potential in the diesel exhaust than in the rapeseed methyl ester (RME), but reported that the results observed were less dramatic in the rat hepatocyte model. They attribute this difference to the differences in metabolic capacities between the models.

The cytotoxic effects of biodiesel exhaust emissions have also been evaluated and compared to petroleum diesel fuel emissions (Bünger et al. 2000). Cytotoxic effects of biodiesel from RME emissions were greater than those of petroleum diesel fuel. Exhaust from rapeseed biodiesel was 4-fold more potent than petroleum diesel exhaust in inducing cytotoxicity (measured as the median effective dose; ED50). Cytotoxicity of biodiesel emissions increased with extract collected with the engine “idling.”

Although it can sometimes be difficult to place the results of animal investigation into the context of potential human health hazard, animal models are viewed to be superior to *in vitro* studies for establishing pulmonary and extrapulmonary responses to potentially toxic exposures (Finch et al. 2002). Subchronic exposure of rats to emissions from a diesel engine burning soybean oil–derived biodiesel fuel induced a dose-related increase in particle-containing alveolar macrophages—a consistent observation in rats subchronically exposed to petroleum diesel exhaust (Finch et al. 2002; Mauderly 1994). The vast majority of exposed rats had little or no evidence of lung neutrophilia and centriacinar fibrosis (Finch et al. 2002). Overall, the study concluded that there were neither significant differences from air exposed control group nor consistent exposure-related significant differences among most of the health parameters evaluated.

To date, only one epidemiologic study has been conducted in which the acute health effects from exposure to biodiesel exhaust fumes were assessed by a questionnaire given to workers who are typically exposed to diesel fumes (e.g., delivery truck drivers, road-maintenance workers, and industrial fork lift truck drivers) (Hasford et al. 1997). This investigation demonstrated dose-dependent respiratory symptoms after exposure to RME and diesel fumes, but there were no significant differences between the two fuels.

## Research Needs Regarding Biodiesel Exhaust Emissions and Human Health

Although currently accounting for a small fraction of diesel use, biodiesel is a fuel alternative that has shown potential for becoming a commercially accepted part of this nation’s energy infrastructure. Biodiesel exhaust emission has been extensively characterized under field and laboratory conditions. Regarding research into any association of biodiesel exhaust exposure and human health end points, there are only a few cytotoxicity and mutagenicity studies. Investigation into an inflammatory and fibrotic response *in vivo* or induction of processes that are good biomarkers of these responses *in vitro* (e.g., cytokine production changes, comprehensive genomic analyses) has not been initiated. Similarly, any interaction of biodiesel exhaust emissions with the atopic state of an individual requires study.

Research institutions focused on public health should use the experience acquired in research focused on petroleum diesel exhaust exposure and human health effects research and perform similar studies with biodiesel exhaust. This research on petroleum diesel exhaust has been summarized and includes epidemiologic studies, animal and human exposure studies, and *in vitro* methodology (U.S. EPA 2002b). As a result of this investigation, petroleum diesel exhaust is recognized to cause a tissue-specific and systemic inflammation and cardiopulmonary injury and aggravate allergic disease.

An immediate need for any research focused on biodiesel exhaust and human health is production and collection of sufficient quantities of exhaust material to be shared by researchers. There are investigators who have isolated various extracts of biodiesel exhaust. These emission extracts have used widely divergent engines, conditions of running, fuels, fuel additives, and after treatments. Frequently, they are generated in quantities of milligrams or less. Under these circumstances, there are limits to both the end points that can be examined and the reproducibility of studies. Furthermore, it is unclear how applicable the results of any single study would be to the field. Availability of large quantities of a particle comparable to the petroleum diesel exhaust particle provided by the National Institute of Standards and Technology would be useful for comparisons between laboratories. Ideally, disparate conditions (on- and off-road vehicles) and different biodiesel fuels can be used under various conditions of running to generate exhaust emissions that could be provided to investigators. Collection strategies should be comparable. Finally, a diesel exhaust particle might also be produced using the same engine and conditions of running for comparison of biologic end points.

In the collection of biodiesel exhaust for establishing a reference material, some thought should be given on how to optimally collect the emission components. The method employed in the testing of biodiesel exhaust may be important in discerning biologic responses. Ideally, for biologic assay, both the gas and particulate phase should be present; the components should be delivered to the biologic assay system in a manner that attempts to reflect the physicochemical composition of the exhaust in an ambient setting with consideration given to factors such as aging, transport, dilution, and potential influences from other factors (e.g., interactions with pollutants). Alternatively, collection and storage of some components of the exhaust, though possibly not in the same form as those emitted, may prove acceptable. Collection devices include filters for primarily particulate matter, liquids (aqueous and/or organic) in impingers (collecting gases and particulate matter with various degrees of efficiencies), use of large inert bags or cylinders to collect a limited volume of the whole exhaust, and resins (to collect volatile and semivolatile components). Filter-based collection systems have historically been used mainly with petroleum diesel exhaust, but the particulate fraction is a minor component emitted, mass-wise (U.S. EPA 2002b). As previously discussed, biodiesel exhaust typically has less particulate matter than does petroleum diesel exhaust, and collection of the particulate fraction may be even more problematic. New and future diesel exhaust control strategies radically reduce the particulate fraction (e.g., > 90% regulated reduction in particulate matter from new on-road and off-road diesel engines (U.S. EPA 2001 e.g., > 90% regulated reduction in particulate matter from new on-road and off-road diesel engines (U.S. EPA 2004). Hence, strategies for the collection of biodiesel and petroleum diesel exhaust should consider alternatives to filter-based systems (e.g., improved electrostatic precipitation techniques may provide a technique to collect primarily the particulate components).

Particles from both combustion and non-combustion processes are associated with an oxidative stress. After exposure to oxidant generation by numerous particles, a cascade of reactions follows in numerous, disparate cell types. This response includes activation of cell signaling, transcription factor activation, and release of proinflammatory mediators. The result is an acute inflammation both in the lower respiratory tract and systemically. This series of reactions may also be evident in response to biodiesel exhaust. This is testable using a myriad of *in vitro* and *in vivo* (animal and human) exposure methodologies. The soluble organic fraction present in petroleum diesel exhaust particles has been associated with both the generation of an oxidative stress and the magnitude of the cytokine response (Bayram et al. 1998; Boland et al. 1999; Bonvallot et al. 2001, 2002; Casillas et al. 1999; Kawasaki et al. 2001). The greater fraction of soluble organic fraction in biodiesel exhaust particles could affect both oxidative stress and the magnitude of the biologic response after exposure to biodiesel exhaust (Graboski and McCormick 1998).

It is our opinion that biodiesel requires greater due diligence than it has received to date in the United States. In widespread employment of biodiesel as an alternative fuel, there would be several additional issues pertinent to human health:

- In the United States, biodiesel is sold as a blend with petroleum diesel [as either B2 (2% biodiesel) or B20 (20%)] and is considered an oxygenate. Such employment is likely to affect the quantity and the composition of emissions and potential biologic effects of the exhaust (Graboski and McCormick 1998). Eventually, research into potential consequences of biodiesel exhaust exposure on human health will have to consider blends.
- Additives to biodiesel fuel are numerous and may affect human health. No emissions data are yet available for biodiesel combined with the additives required for practical application of biodiesel fuel use on a national level. These include cetane improvers, smoke suppressors, flow enhancers, cloud-point depressors, wax antisettling additives, and detergents to reduce injector nozzle fouling, antioxidants for unsaturated oils, and control of microbial growth. Some of these additives include metals. Ideally, their participation in the biologic effects of exposure to biodiesel exhaust should be tested.
- Disparate levels of aldehydes in biodiesel fuel and its exhaust emissions may be associated with varying impacts on indices of human health. Low-quality biodiesel, which does not meet high production standards, will emit greater quantities of aldehydes because of poor post-transesterification refining. Studies also demonstrate that emissions from RME may have elevated concentrations of aldehydes relative to those from soybean methyl ester. It is unclear whether this might affect human health (Krahl et al. 2001).
- Implementation of new petroleum diesel engine combustion and after-treatment technologies, designed to decrease specific exhaust components such as particulate matter and nitrogen oxides, will require re-evaluation of the emissions from biodiesel exhaust. The decrease in specific components is being driven partly by integration of new regulations and standards for on-road heavy-duty diesel engines and off-road regulations stretching out to 2016. Similar legislation regarding stricter emission standards from on-road heavy-duty diesel engines are being implemented in the European Union. However, it is not clear whether decreased emission rates of some targeted components (e.g., nitrogen oxides) decrease all health-related end points. In one report, a new diesel particulate trap technology can increase emissions of some toxic components (Ullman et al. 2003).
- The use of pesticides on plants with subsequent contamination of feedstocks could possibly affect specific end points of human health and requires further investigation (Van Gerpen et al. 2004).

Currently there is a strong desire and need for alternative fuels in this country. Employment of biodiesel fuel is favorably viewed, and there are suggestions that its exhaust emissions are less likely to present any risk to human health relative to petroleum diesel emissions (Mauderly 1997). However, the speculative nature of a reduction in health effects based on chemical composition of biodiesel exhaust needs to be followed up with investigations using newer biologic approaches gained from years of diesel research. Studies into health effects of exposure to biodiesel exhaust should be initiated.

## Figures and Tables

**Figure 1:**
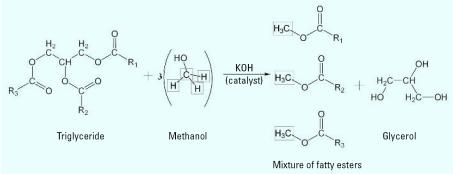
Transesterification reaction for producing biodiesel from triglycerides where R1, R2, and R3 are long chains of saturated/unsaturated hydrocarbons (i.e., fatty-acid chains). Typically potassium hydroxide (KOH) is used as the catalyst.

**Figure 2:**
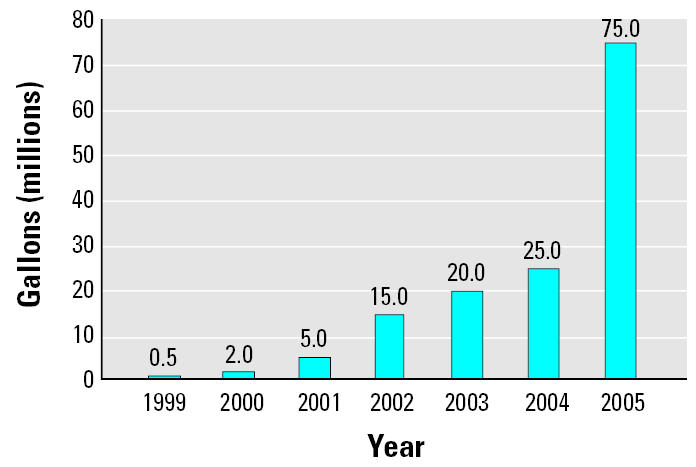
Estimated U.S. biodiesel production per year. Data from National Biodiesel Board (2005).
